# Smartphone Cognitive Behavioral Therapy as an Adjunct to Pharmacotherapy for Refractory Depression: Randomized Controlled Trial

**DOI:** 10.2196/jmir.8602

**Published:** 2017-11-03

**Authors:** Akio Mantani, Tadashi Kato, Toshi A Furukawa, Masaru Horikoshi, Hissei Imai, Takahiro Hiroe, Bun Chino, Tadashi Funayama, Naohiro Yonemoto, Qi Zhou, Nao Kawanishi

**Affiliations:** ^1^ Mantani Mental Clinic Hiroshima Japan; ^2^ Aratama Kokorono Clinic Nagoya Japan; ^3^ Department of Health Promotion and Human Behavior School of Public Health, Graduate School of Medicine Kyoto University Kyoto Japan; ^4^ National Center of Neurology and Psychiatry Kodaira Japan; ^5^ Waseda Clinic Kani Japan; ^6^ Ginza Taimei Clinic Tokyo Japan; ^7^ Funayama Mental Clinic Nagoya Japan; ^8^ Department of Biostatistics School of Public Health, Graduate School of Medicine Kyoto University Kyoto Japan; ^9^ Department of Health Research Methods, Evidence, and Impact McMaster University Hamilton, ON Canada; ^10^ Advanced Telecommunications Research Institute International Kyoto Japan

**Keywords:** major depressive disorder, pharmacotherapy-resistant depression, mobile phone app, cognitive behavioral therapy, eHealth

## Abstract

**Background:**

In the treatment of major depression, antidepressants are effective but not curative. Cognitive behavioral therapy (CBT) is also effective, alone or in combination with pharmacotherapy, but accessibility is a problem.

**Objective:**

The aim is to evaluate the effectiveness of a smartphone CBT app as adjunctive therapy among patients with antidepressant-resistant major depression.

**Methods:**

A multisite, assessor-masked, parallel-group randomized controlled trial was conducted in 20 psychiatric clinics and hospitals in Japan. Participants were eligible if they had a primary diagnosis of major depression and were antidepressant-refractory after taking one or more antidepressants at an adequate dosage for four or more weeks. After a 1-week run-in in which participants started the medication switch and had access to the welcome session of the app, patients were randomized to medication switch alone or to medication switch plus smartphone CBT app via the centralized Web system. The smartphone app, called Kokoro-app (“kokoro” means “mind” in Japanese), included sessions on self-monitoring, behavioral activation, and cognitive restructuring presented by cartoon characters. The primary outcome was depression severity as assessed by masked telephone assessors with the Patient Health Questionnaire-9 (PHQ-9) at week 9. The secondary outcomes included the Beck Depression Inventory-II (BDI-II) and Frequency, Intensity, and Burden of Side Effects Ratings (FIBSER).

**Results:**

In the total sample (N=164), 81 participants were allocated to the smartphone CBT in addition to medication change and 83 to medication change alone. In the former group, all but one participant (80/81, 99%) completed at least half, and 71 (88%) completed at least six of eight sessions. In the intention-to-treat analysis, patients allocated the CBT app scored 2.48 points (95% CI 1.23-3.72, *P*<.001; standardized mean difference 0.40) lower on PHQ-9 than the control at week 9. The former group also scored 4.1 points (95% CI 1.5-6.6, *P*=.002) lower on BDI-II and 0.76 points (95% CI –0.05 to 1.58, *P*=.07) lower on FIBSER. In the per-protocol sample (comfortable with the smartphone app, still symptomatic, and adherent to medication with mild or less side effects after run-in), the intervention group (n=60) scored 1.72 points (95% CI 0.25-3.18, *P*=.02) lower on PHQ-9, 3.2 points (95% CI –0.01 to 6.3, *P*=.05) lower on BDI-II, and 0.75 points (95% CI 0.03-1.47, *P*=.04) lower on FIBSER than the control (n=57). The treatment benefits were maintained up to week 17.

**Conclusions:**

This is the first study to demonstrate the effectiveness of a smartphone CBT in the treatment of clinically diagnosed depression. Given the merits of the mobile mental health intervention, including accessibility, affordability, quality control, and effectiveness, it is clinically worthwhile to consider adjunctive use of a smartphone CBT app when treating patients with antidepressant-resistant depression. Research into its effectiveness in wider clinical contexts is warranted.

**Trial Registration:**

Japanese Clinical Trials Registry UMIN CTR 000013693; https://upload.umin.ac.jp/cgi-open-bin/ctr_e/ ctr_view.cgi?recptno=R000015984 (Archived by WebCite at http://www.webcitation.org/6u6pxVwik)

## Introduction

Major depression is highly prevalent, debilitating, and costly [1-3]. It is predicted to be the leading cause of premature mortality and disability in high-income countries by 2030, and the third-leading cause in low- and middle-income countries [4]. Resources for and access to care by those who suffer remain constrained in high-income countries, and severely so in low- to middle-income ones [5,6].

Although antidepressant pharmacotherapy represents the mainstay of treatment of major depression [7], after several weeks of treatment only 50% show reduction by half or more in their depression severity and only 30% return to a euthymic state [8]. No standard approach in the management of treatment-refractory depression exists. Guideline recommendations include increasing the dose, switching to another antidepressant, or augmenting treatment with another pharmacological or psychological therapy [9,10].

Cognitive behavioral therapy (CBT) has proved an effective treatment of major depression either alone [11] or in combination with pharmacotherapy [12,13]. A standard course of CBT, however, requires 10 to 20 sessions, each lasting 45 to 60 minutes, with an adequately qualified professional. Therefore, its availability is limited everywhere in the world [14,15]. Telephone or videoconference CBT eliminates the burden of in-person visits, but requires approximately equal therapist time and equal competence.

Guided self-help CBT requires minimal to no clinician time and has proved of comparable effectiveness to its face-to-face versions [16]. Information and communication technologies (ICT) for self-help CBT, including computerized stand-alone software and Internet-based webpages, have shown promising results in initial trials [17]. However, one recent pragmatic trial using two widely known online Web-based CBT packages failed to demonstrate added value over usual care in primary care likely because adherence to the program was very low [18].

The dominant modality of ICT is evolving rapidly and the smartphone is now transforming people’s lives across the world. In comparison with stand-alone or Internet-connected computers, the smartphone enhances portability and immediacy, making CBT fully accessible and therefore promising new dimensions of guided self-help. Mobile health apps are currently proliferating in the electronic world, with more than 165,000 health apps available online [19-21]. However, few apps have demonstrated quality [22,23] and no randomized controlled trial (RCT) has yet proved the benefits of a smartphone app in comparison with a control condition in the treatment of clinically diagnosed major depressive disorder. Several trials have examined the use of smartphone to monitor symptoms [24-26] or smartphone apps applying the CBT principles, but only among participants recruited from the general population who had reported elevated levels of depressive symptoms on self-reports [27-33]. A few have examined participants with diagnosed major depression, but only against active controls. One pilot RCT compared the original Web-based CBT against its smartphone version in 35 participants with depression confirmed through telephone diagnostic interview [34]. Another group of researchers developed a smartphone app for behavioral activation and tested it against its face-to-face full version [35] or against a mindfulness-based program [36] among 93 and 81 patients, respectively, who were recruited through advertisements in mass media but whose diagnosis of major depression was confirmed through telephone interview. In these studies, results did not differ significantly between the intervention and control groups.

Whether smartphone-based CBT can have any demonstrable value in the treatment of clinically diagnosed major depression is a pressing issue for patients, clinicians, and policy makers around the world [22,23]. We have developed and pilot-tested a smartphone app, called Kokoro-app (*kokoro* means “mind” in Japanese), that is based on a CBT manual with demonstrated effectiveness in remote telephone or group formats in several RCTs either alone [37,38] or in combination with antidepressants [39]. This study represents the first RCT to examine adjunctive smartphone-based CBT to medication change among patients with major depression unresponsive to prior antidepressant treatments.

## Methods

### Design

The study was a multisite, assessor-masked, parallel-group RCT with a 1:1 allocation ratio.

### Participants

A detailed description of the study protocol has been reported elsewhere [40] and is attached as Multimedia Appendix 1. The study, which was approved by the ethics committees of the participating centers and registered in the Japanese clinical trials registry (UMIN CTR 000013693), took place in 20 psychiatric clinics and hospitals across Japan between September 2014 and October 2016. Multimedia Appendix 2 provides the complete list of trial sites and investigators.

Eligible participants (1) were aged between 25 and 59 years, (2) had a primary diagnosis of major depressive disorder without psychotic features according to the *Diagnostic and Statistical Manual of Mental Disorders* (Fifth Edition) [41] as ascertained by using the Primary Care Evaluation of Mental Disorders procedure [42], (3) were antidepressant-resistant, defined as scoring 10 or more on the Beck Depression Inventory-II (BDI-II) [37,43] after taking one or more antidepressants at an adequate dosage for four or more weeks (stage I, II, or III according to the criteria by Thase and Rush [44]), (4) had not been prescribed escitalopram or sertraline, or received CBT or interpersonal therapy for the index episode.

The study psychiatrists introduced the trial to the potentially eligible patients from among the patients they were seeing and invited them to participate voluntarily. After full disclosure of the trial contents and procedures, all participants provided written informed consent. No advertisement through the media was used.

### Randomization

On entry, all participants started switching their antidepressant, had the Kokoro-app installed onto their iPhone, and had access to the welcome session, which mainly aimed at, after a brief description of CBT, training the participants in the use of the smartphone and its speech recognition. (When the participants did not own an iPhone, we lent one to them for the duration of the trial.) After this 1-week run-in, they had a telephone interview with the Patient Health Questionnaire-9 (PHQ-9) and the Frequency, Intensity, and Burden of Side Effects Ratings (FIBSER) and were classified into one of two samples. The per-protocol sample comprised those who had not or only partially responded since week 0 (PHQ-9 scores ≥5 [45] at week 1), who tolerated and adhered to the new antidepressant, and had no problem using the smartphone and Kokoro-app. The auxiliary sample included all others. The total sample (the per-protocol sample plus the auxiliary sample) would answer the real-world question of the value of smartphone CBT among the patients for whom the clinicians would initially consider prescribing the smartphone CBT, including those who would not tolerate the new medication or who may have some difficulty using the smartphone app, whereas the per-protocol sample would answer the question of the value of smartphone CBT under the narrower circumstances (ie, among patients who were able to follow the protocol and for whom the app would be expected to demonstrate its full effects).

After stratification by group, the participants were randomized 1:1 to the combined antidepressant switch plus smartphone CBT arm (intervention arm) or the antidepressant switch alone arm (control arm) using an automated Web program implementing the method of minimization. Therefore, the randomization was concealed. Clinics, number of antidepressants previously prescribed for the index episode (≥3 vs <3), and a total score of the PHQ-9 at week 1 (≥10 vs <10) were used as minimization variables.

During the telephone interview for the PHQ-9, if participants reported suicidal ideation for more than half the days or nearly every day of the previous 2 weeks, the interviewer immediately notified the staff in the central office, who then notified the responsible psychiatrist.

### Interventions

The intervention group received both the antidepressant switch and the smartphone CBT, whereas the control group received only the antidepressant switch during the intervention period up to week 9. Details of each intervention are described subsequently.

#### Antidepressant Switch

All study participants started switching their antidepressant either to escitalopram (5-10 mg/day) or to sertraline (25-100 mg/day) at week 0. The previous antidepressant was tapered off by week 5. We limited choice of antidepressants to escitalopram and sertraline, which showed a favorable profile in efficacy and acceptability in a previous systematic review [46], to ensure balance in antidepressant treatments during the trial. If the participants did not tolerate escitalopram or sertraline, the physician could revert to the previous antidepressant or start a new one. Only anxiolytics and hypnotics were allowed as coprescribed psychotropics. The frequency of visit was set at least once in four weeks, with additional visits as judged necessary by the study physician.

#### Smartphone Cognitive Behavioral Therapy

**Figure 1:**
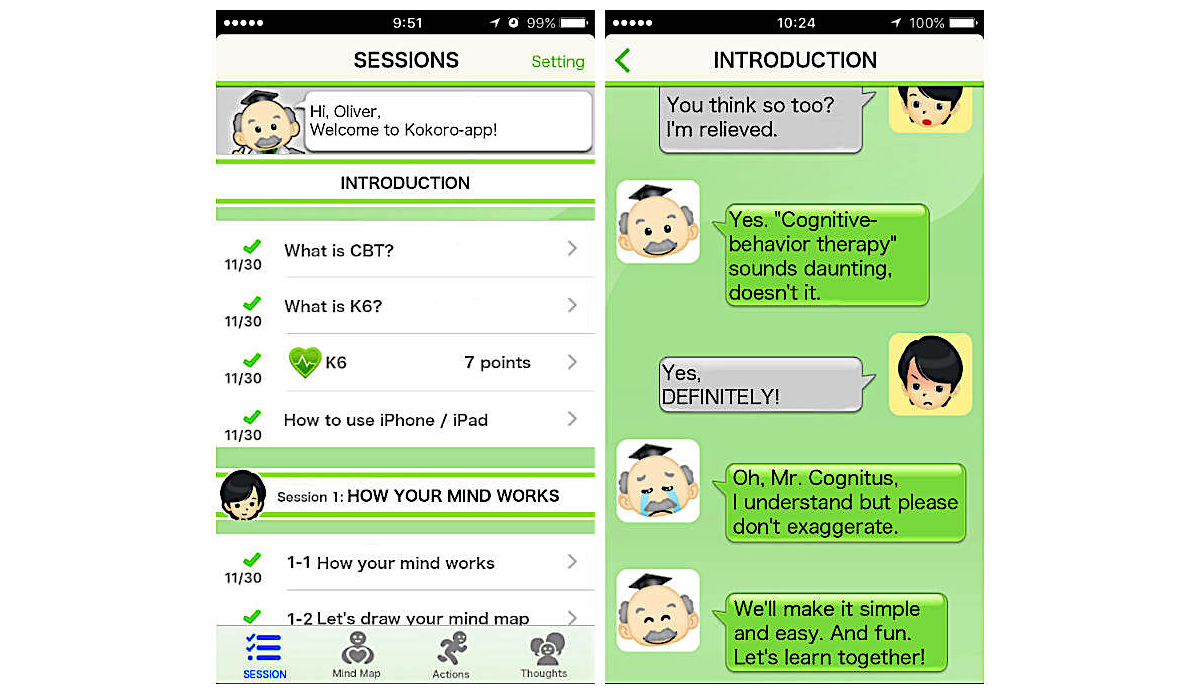
Screenshots from Kokoro-app.

Kokoro-app is a self-help smartphone app consisting of eight sessions, including one welcome session, two sessions on self-monitoring, two sessions on behavioral activation, two sessions on cognitive restructuring, and an epilog focusing on relapse prevention. In each session, explanation of the principles and skills of CBT is provided in the format of instant messenger exchanges among cartoon characters (Figure 1). In the self-monitoring sessions, patients learn how to monitor their reactions to situations in terms of feelings, thoughts, body reactions, and behaviors and describe them in “mind maps.” In the behavioral activation sessions, patients learn to engage in “personal experiments” of small pleasurable actions according to the principle “When your body moves, so does your mind.”

To help patients broaden their thoughts, the cognitive restructuring sessions provide four tools, each of which guides the patients to alternative thoughts through interaction with the characters. Each session is supposed to take 1 week: participants can proceed to the next session only after 1 week and only after they have completed one homework from that session. See Multimedia Appendix 3 for more details. One session requires approximately 20 minutes to complete.

The progress of each participant on Kokoro-app could be monitored at “Kokoro-Web.” Participants and their treating psychiatrists could access their records using a unique identification number and password. Secure Sockets Layer certified security of the data exchanged through the Internet.

Every week, the central office sent an email to participants to congratulate them on their progress. The message was templated but individually modified based on the progress of participants and the comments they uploaded at the end of each session. Writing one such email took 3 to 10 minutes.

All study participants had access to the welcome session at week 0. After randomization at week 1, the intervention group received another password and continued with further sessions. The control group continued with the medication change only. Face-to-face CBT or interpersonal therapy were prohibited for either group.

#### Follow-Up Period After 9 Weeks

After assessments at week 9, there were no further restrictions in medications or psychotherapies. The participants in the control arm received a password to begin the Kokoro-app; follow-up assessments took place at week 17.

### Outcomes

The masked assessors interviewed the participants by telephone with PHQ-9 and FIBSER at weeks 0, 1, 5, 9, and 17.

The primary outcome was the PHQ-9 at week 9. It consists of the nine diagnostic criteria items of major depression [47]. Each item is rated between 0=“not at all” through 3=“nearly every day.” The total score ranges between 0 and 27. The instrument has excellent reliability, validity, and responsiveness [48]. Remission was defined as scoring four or less on PHQ-9, and response as 50% or greater reduction from baseline.

Secondary outcomes included the FIBSER, which assesses the frequency, intensity, and burden of side effects, each on a 7-point scale, with a total score between 3 and 27, [49] and the BDI-II [43], a self-report measure of depression severity that asks about 21 symptoms of depression, each on a scale between 0 and 3, with the total score between 0 and 63.

The study participants and the psychiatrists in charge of medication change were aware of the treatment allocation. The outcome assessors conducting telephone interviews were unaware of allocation. The success of this masking was evaluated by calculating the Bang Index [50] of assessors’ treatment guesses after each telephone assessment. The Bang Index is scaled to an interval of –1 to 1, 1 being complete lack of blinding, 0 being consistent with perfect random guessing, and –1 indicating opposite guessing.

### Sample Size

The study was powered to detect a moderate effect size of 0.5 in terms of standardized mean difference between the two treatments for the primary outcome at week 9, with 80% power at two-sided alpha level of .05. Assuming that 30% would leave the study or be classified into the auxiliary group at week 1, the required total sample size was 164 participants.

### Statistical Analysis

All analyses were undertaken according to the intention-to-treat principle, including all the randomized participants for the total sample as well as for the per-protocol sample.

For each continuous outcome up to week 9, we used a linear mixed model including sites and patients as random effects and time (5 and 9 weeks), treatment, and time*treatment interaction, adjusting for its baseline score and the stratification variables, as fixed effects. The primary endpoint was the estimate of the least squares mean difference along with the 95% confidence interval (95% CI) at week 9. For the continuous outcome at week 17 follow-up, we used the similar linear mixed model but without the time*treatment interaction because this was a one-time comparison after all the participants received the smartphone CBT both in the intervention and the control groups. For the dichotomous outcomes, we used a generalized linear mixed model with the same random effects and the fixed effects. We chose odds ratios as the measure of effect. We used SAS version 9.4 (SAS Institute Inc, Cary, NC, USA). Multimedia Appendix 1 provides the statistical analysis plan.

### Blinded Interpretation of the Results

The statistician (QZ), blinded to allocation, conducted the statistical analyses. The writing committee reviewed a statistical report in which the two treatment arms were designated A and B, and developed interpretation of the results and associated conclusions under two different scenarios, one assuming A to be the smartphone CBT plus medication change arm and B to be the medication change alone arm, and another alternative scenario. The code was broken only after the writing committee signed off on the agreed-on interpretations (see Multimedia Appendix 4).

## Results

### Participant Characteristics

**Figure 2:**
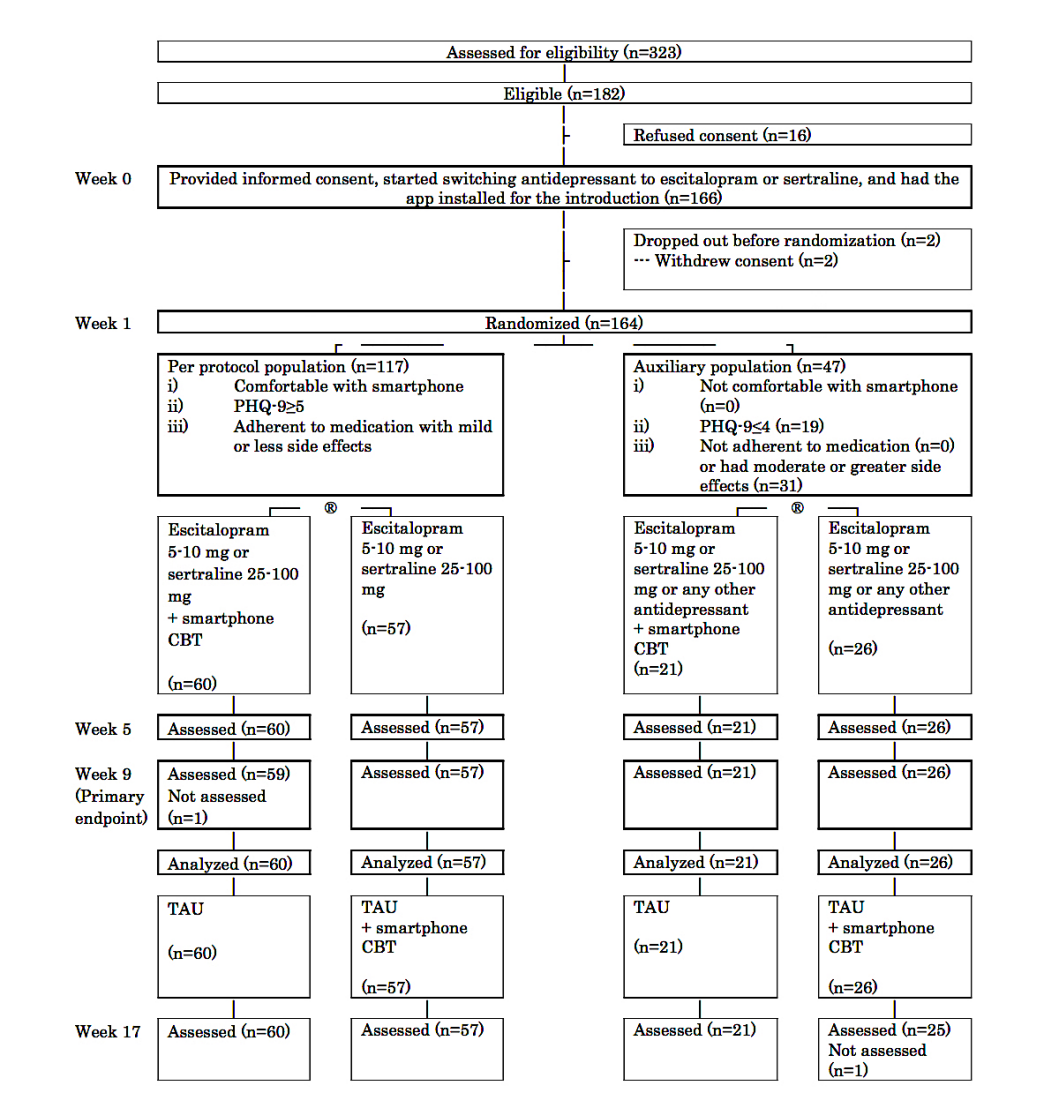
Assessment, randomization, and follow-up of study participants. CBT: cognitive behavioral therapy; PHQ-9: Patient Health Questionnaire 9; TAU: treatment as usual.

Figure 2 shows the flow of participants through the study. Between September 2, 2014, and July 1, 2016, 323 patients were assessed for eligibility; 166 patients provided informed consent and started medication change. Two withdrew consent before randomization at week 1. Therefore, we recruited 164 patients, of whom 117 found no difficulty with the smartphone, were adherent to the protocol treatment, remained at least moderately symptomatic, and constituted the per-protocol sample; 60 were allocated to the smartphone CBT and 57 to medication change alone. Of the remaining 47 participants in the auxiliary group, 21 were allocated to the intervention group and 26 to control. Primary outcome data at 9 weeks were obtained from all but one randomized participant (163/164, 99.4%).

Table 1 shows that the baseline demographic and clinical characteristics of the treatment groups were well balanced. Typically, patients were in their thirties to forties, had three previous episodes, were in the current episode for nearly 2 years, and were severely to moderately depressed.

**Table 1:** Baseline characteristics of the intention-to-treat samples.

Characteristics			Total sample (N=164)		Per-protocol sample (n=117)	
			Smartphone CBT + medication change (n=81)	Medication change alone (n=83)	Smartphone CBT + medication change (n=60)	Medication change alone (n=57)
**Demographic characteristics**						
	Age (years), mean (SD)		40.2 (8.8)	41.6 (8.9)	40.1 (9.0)	41.2 (8.6)
	Sex (female), n (%)		46 (57)	41 (50)	35 (58)	31 (54)
	Education (years), mean (SD)		14.6 (2.5)	14.9 (2.7)	14.8 (2.5)	15.1 (2.9)
	**Employment status, n (%)**					
		Employed full-time	34 (42)	29 (35)	27 (45)	21 (37)
		Employed part-mime	7 (9)	5 (6)	5 (8)	5 (9)
		On medical leave	21 (26)	30 (36)	14 (23)	21 (37)
		Housewife	6 (7)	5 (6)	5 (8)	3 (5)
		Student	0	2 (21)	0	1 (2)
		Retired	0	0	0	0
		Not employed	13 (16)	12 (15)	9 (15)	6 (11)
	**Marital status, n (%)**					
		Single, never married	34 (42)	31 (38)	24 (40)	22 (39)
		Single, divorced, separated or widowed	13 (16)	7 (8)	12 (20)	5 (9)
		Married	34 (42)	45 (54)	24 (40)	30 (53)
**Baseline clinical characteristics, mean (SD)**						
	Age of onset at first episode (years)		31.8 (10.8)	34.6 (10.0)	32.2 (11.0)	34.0 (10.2)
	Number of previous depressive episodes		3.4 (4.9)	3.0 (4.5)	3.5 (4.7)	2.8 (4.6)
	Length of current episode (months)		24.2 (46.3)	23.0 (46.5)	27.0 (52.6)	22.3 (42.5)
	**PHQ-9**^a^					
		Week 0	13.5 (5.5)	12.9 (5.3)	14.0 (5.2)	13.8 (5.1)
		Week 1	12.6 (6.2)	11.9 (5.9)	13.4 (5.6)	12.6 (5.5)
	**BDI-II**^a^					
		Week 0	28.2 (11.2)	26.2 (11.0)	29.4 (10.6)	27.4 (10.7)
		Week 1	26.2 (11.7)	24.7 (12.2)	28.1 (11.0)	26.2 (11.6)
	**FIBSER**^a^					
		Week 0	4.8 (4.5)	5.2 (3.1)	4.4 (2.8)	5.4 (2.9)
		Week 1	6.4 (4.5)	6.8 (4.4)	5.0 (3.0)	5.2 (2.5)

^a^BDI-II: Beck Depression Inventory-II; FIBSER: Frequency, Intensity, and Burden of Side Effects Ratings; PHQ-9: Patient Health Questionnaire 9.

### Treatments Received

In the total sample, 16 of 81 participants (20%) stopped the protocol antidepressant treatment by escitalopram or sertraline in the intervention arm, as did 14 of 83 (17%) in the medication only arm (Table 2). Some received augmentation drugs, such as antipsychotics or lithium, which were not allowed in the protocol for the antidepressant switch (n=15 in the total sample), a few had deterioration or side effects and could not continue the protocol treatment (n=5), and a few others got so well and did not want to continue with the protocol medication (n=2). The medication dosages were comparable between the arms.

For the smartphone CBT, all but one participant (80/81, 99%) completed at least half the sessions of the program, and 71 of 81 (88%) completed at least six of eight sessions. It took the patients, on average, 10.8 (SD 4.2) days to complete one session. The patients filled in a mean 11.2 (SD 11.4) “mind maps” for self-monitoring, conducted a mean 14.4 (SD 17.1) behavioral activation “personal experiments,” and generated a mean 6.1 (SD 6.0) alternative thoughts for cognitive restructuring (Table 2).

**Table 2:** Treatment received in the intention-to-treat samples.

Therapy			Total sample (N=164)		Per-protocol sample (n=117)	
			Smartphone CBT + medication change (n=81)	Medication change alone (n=83)	Smartphone CBT + medication change (n=60)	Medication change alone (n=57)
**Pharmacotherapy**						
	Discontinuation of protocol antidepressant treatment by escitalopram or sertraline by week 9, n (%)		16 (20)	14 (17)	10 (17)	8 (14)
	**Discontinuation reason, n**					
		Prescription of prohibited drugs	6	9	4	5
		Amelioration	1	1	—	1
		Deterioration	1	—	1	—
		Side effects	3	1	1	—
		Other	5	3	4	2
	Discontinuation of any antidepressant therapy by week 9, n (%)		5 (6)	2 (2)	2 (3)	2 (4)
	Escitalopram dosage at week 9 (mg/day), mean (SD)		9.5 (3.0), n=48	10.0 (3.2), n=49	9.7 (3.1), n=38	10.1 (2.9), n=34
	Sertraline dosage at week 9 (mg/day), mean (SD)		79.0 (26.7), n=25	83.6 (23.4), n=29	81.6 (26.1), n=19	83.0 (24.9), n=22
**Smartphone CBT**						
	**Sessions completed, n**			—		—
		0	1		—	
		3	1		1	
		4	4		3	
		5	4		2	
		6	11		8	
		7	17		13	
		8	43		33	
	Time per session (days), mean (SD)		10.8 (4.2)	—	10.7 (4.0)	—
	Number of mind maps for self-monitoring, mean (SD)		11.2 (11.4)	—	10.8 (10.3)	—
	Number of behavioral activation tasks, mean (SD)		14.4 (17.1)	—	14.6 (17.8)	—
	Number of alternative thoughts, mean (SD)		6.1 (6.0)	—	6.5 (6.0)	—

**Table 3:** Outcomes at weeks 9 and 17 for the total sample (N=164).

Outcomes^a^		Smartphone CBT + medication change (n=81), mean/% (95% CI)	Medication change alone (n=83), mean/% (95% CI)	Adjusted difference/OR (95% CI)^b^	*P* value
**End of randomized trial (week 9)**					
	PHQ-9	7.94 (6.98, 8.89)	10.41 (9.45, 11.33)	–2.48 (–3.72, –1.23)	<.001
	Remission	30.5% (19.7%, 43.9%)	17.8% (10.3%, 29.0%)	2.02 (0.93, 4.42)	.08
	Response	42.3% (29.4%, 56.4%)	21.2% (12.7%, 33.2%)	2.73 (1.35, 5.53)	.005
	BDI-II	19.3 (17.0, 21.5)	23.3 (21.6, 25.5)	–4.1 (–6.6, –1.5)	.002
	FIBSER	4.38 (3.72, 5.03)	5.14 (4.52, 5.76)	–0.76 (–1.58, 0.05)	.07
**Follow-up (week 17)**					
	PHQ-9	7.95 (6.73, 9.17)	8.76 (7.58, 9.95)	–0.81 (–2.24, 0.62)	.26
	BDI-II	17.2 (14.4, 20.0)	19.1 (16.4, 21.8)	–1.9 (–4.9, 1.2)	.22
	FIBSER	4.62 (3.83, 5.42)	5.10 (4.32, 5.89)	–0.48 (–1.34, 0.37)	.27

^a^BDI-II: Beck Depression Inventory-II, FIBSER: Frequency, Intensity, and Burden of Side Effects Ratings, PHQ-9: Patient Health Questionnaire 9.

^b^For each continuous outcome up to week 9, we used a linear mixed model including sites and patients as random effects and time (5 and 9 weeks), treatment, and time*treatment interaction, adjusting for its baseline score and the stratification variables, as fixed effects. For the continuous outcome at week 17 follow-up, we used the similar linear mixed model but without time*treatment interaction. For the dichotomous outcomes, the generalized linear mixed model was used along with the same random effects and the fixed effects. The summary effect measures are adjusted score differences for PHQ-9, BDI-II, and FIBSER, and are odds ratios for remission and response.

### Outcomes for the Total Sample

**Figure 3:**
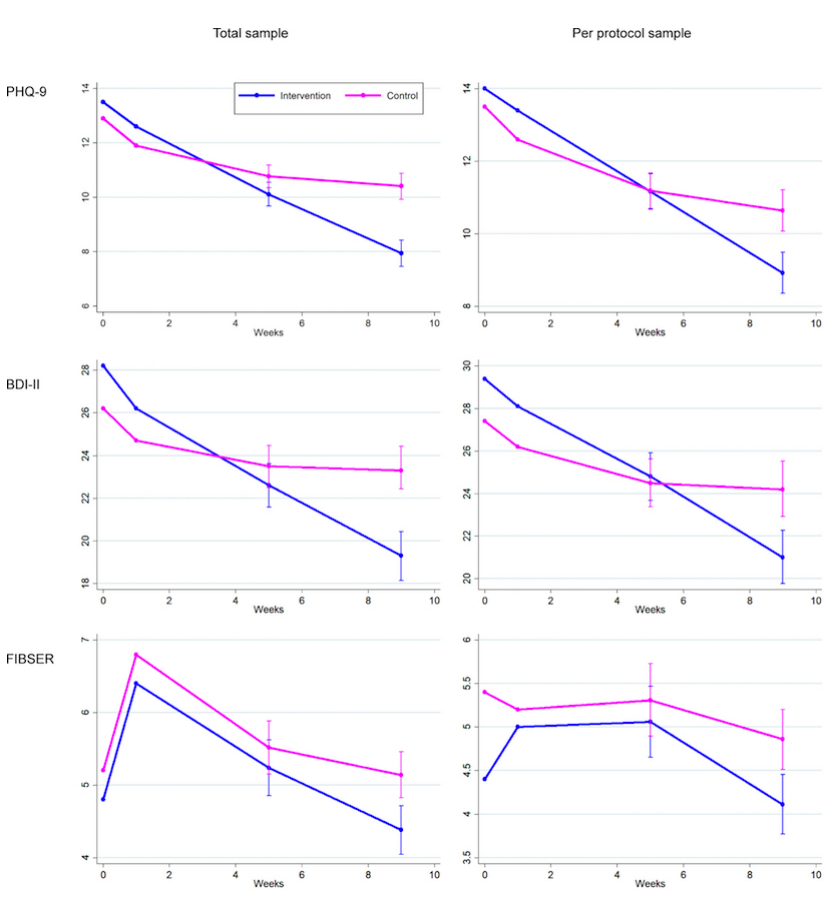
Trajectory of the Patient Health Questionnaire-9 (PHQ-9), Beck Depression Inventory-II (BDI-II), and Frequency, Intensity, and Burden of Side Effects Ratings (FIBSER) scores for the intervention (blue line) and control (red line) groups in the total (N=164) and per-protocol (n=117) samples. Error bars show standard errors for model-based least squares means.

In the intention-to-treat analysis of the total sample, patients who were allocated the CBT app (n=81) scored 2.48 points (95% CI 1.23-3.72, *P*<.001; standardized mean difference [SMD] 0.40) lower on PHQ-9 than those who were not (n=83) at week 9 (Table 3, Figure 3). The former group also scored 4.1 points (95% CI 1.5-6.6, *P*=.002) lower on BDI-II. Significantly more participants showed response (OR 2.73, 95% CI 1.35-5.53, *P*=.005). However, the increase in remission did not reach statistical significance (OR 2.02, 95% CI 0.93-4.42, *P*=.08).

With regard to the harm outcomes, patients using the smartphone CBT reported somewhat less overall burden of side effects, but the difference was not statistically significant (FIBSER mean difference=–0.76, 95% CI –1.58 to 0.05, *P*=.07). There was one report of suicidality (self-injurious behavior without suicidal intent) in the combined treatment arm and one report of a serious adverse event in the control arm (brief hospital admission for examination of preexisting spinal canal stenosis).

### Outcomes for the Per-Protocol Sample

In the per-protocol sample, who were comfortable with the smartphone app, were still symptomatic, and were adherent to medication with mild or less side effects after the run-in, the patients who received smartphone CBT in addition to medication change (n=60) scored mean 1.72 (95% CI 0.25-3.18, *P*=.02; SMD 0.28) points lower on PHQ-9 than those undergoing medication change alone (n=57) at week 9. The combined treatment arm was superior to the control arm in terms of BDI-II (difference=–3.2, 95% CI –6.3 to 0.0, *P*=.05, not statistically significant), but not in terms of remission (OR 1.99, 95% CI 0.74-5.38, *P*=.17) or response (OR 2.11, 95% CI 0.92-4.85, *P*=.08).

In terms of the harm outcome, the combination treatment arm reported significantly less overall burden of side effects (FIBSER mean difference=–0.75, 95% CI –1.47 to –0.03, *P*=.04) (Table 4, Figure 3).

### Follow-Up at Week 17

The participants who had access to the smartphone app in the first 9 weeks maintained their gains for a further 8 weeks. At week 17, when the participants in the control arm also had access to the smartphone app, the results were comparable between the two groups in terms of PHQ-9, BDI-II, and FIBSER, both for the total sample and for the per-protocol sample (Tables 3 and 4).

### Masking

The Bang Index of the treatment guesses by the masked assessors at weeks 5, 9, and 17 was 0.10 (95% CI –0.13 to 0.33), 0.29 (95% CI 0.06-0.51), and 0.30 (95% CI 0.08-0.52) for the intervention arm and –0.21 (95% CI –0.45 to 0.03), –0.18 (95% CI –0.41 to 0.06), and –0.30 (95% CI –0.53 to –0.07) for the control arm, respectively. The observed patterns indicate that the raters guessed the treatment allocation haphazardly or guessed it to be the smartphone CBT arm more often regardless of the actual allocation, resulting in ideally unbiased assessment of outcomes in the trial [51].

**Table 4:** Outcomes at weeks 9 and 17 for the per-protocol sample (n=117).

Outcomes^a^		Smartphone CBT + medication change (n=60), mean/% (95% CI)	Medication change alone (n=57), mean/% (95% CI)	Adjusted difference/OR (95% CI)^b^	*P* value
**End of randomized trial (week 9)**					
	PHQ-9	8.92 (7.81, 10.03)	10.64 (9.52, 11.76)	–1.72 (–3.18, –0.25)^c^	.02
	Remission	18.2% (8.5%, 34.8%)	10.0% (4.0%, 23.2%)	1.99 (0.74, 5.38)	.17
	Response	31.6% (18.7%, 48.3%)	18.0% (9.2%, 32.3%)	2.11 (0.92, 4.85)	.08
	BDI-II	21.0 (18.6, 23.5)	24.2 (21.7, 26.8)	–3.2 (–6.3, 0.0)	.05
	FIBSER	4.11 (3.44, 4.78)	4.86 (4.18, 5.53)	–0.75 (–1.47, –0.03)	.04
**Follow-up (week 17)**					
	PHQ-9	8.92 (7.40, 10.44)	8.85 (7.31, 10.39)	0.07 (–1.68, 1.82)	.94
	BDI-II	19.4 (15.9, 22.9)	20.0 (16.5, 23.6)	–0.6 (–4.4, 3.1)	.75
	FIBSER	4.14 (3.40, 4.88)	4.46 (3.71, 5.21)	–0.32 (–1.28, 0.63)	.50

^a^BDI-II: Beck Depression Inventory-II; FIBSER: Frequency, Intensity, and Burden of Side Effects Ratings; PHQ-9: Patient Health Questionnaire 9.

^b^For each continuous outcome up to week 9, we used a linear mixed model including sites and patients as random effects and time (5 and 9 weeks), treatment, and time*treatment interaction, adjusting for its baseline score and the stratification variables, as fixed effects. For the continuous outcome at week 17 follow-up, we used the similar linear mixed model but without time*treatment interaction. For the dichotomous outcomes, the generalized linear mixed model was used along with the same random effects and the fixed effects. The summary effect measures are adjusted score differences for PHQ-9, BDI-II, and FIBSER, and are odds ratios for remission and response.

^c^Primary endpoint per protocol.

## Discussion

In patients with major depression who had not responded to one or more antidepressants, adding smartphone CBT to medication change was more effective than treatment by medication change alone. The smartphone CBT also decreased the overall side effect burden of the pharmacotherapy.

The magnitude of benefit of the adjunctive mobile CBT was approximately 2 points on the PHQ-9 and 3 to 4 points on the BDI-II. Using the observed standard deviation at week 9, these differences translate into standardized mean differences of 0.28 to 0.40 and are comparable to that of 0.31 for antidepressants over placebo reported in a comprehensive systematic review of phase II or III RCTs [52]. The remission and response rates almost doubled, corresponding with numbers needed to treat between 5 and 12.

Although several smartphone CBT apps have been experimented among general population participants with elevated self-reported symptoms [27-33], Kokoro-app is the first smartphone CBT app to prove to be effective in a RCT in comparison with an alternative treatment for patients suffering from clinically diagnosed major depression. Advantages of smartphone CBT include high accessibility, efficiency, and affordability. Further, it is less susceptible to quality control problems that may plague face-to-face therapies [53].

Unexpectedly, Kokoro-app also reduced the global burden of side effects due to pharmacotherapy. We speculate that the smartphone CBT, through which the patient actively searches for ways to overcome their depression, may increase their sense of self-control and decrease the subjective burden of side effects in comparison with standard pharmacotherapy.

All benefits were larger in the total sample than in the per-protocol comparison, where we had anticipated a larger effect at the protocol stage. Participants were not included in the per-protocol sample mainly because they suffered from side effects from the medication change (31 of 47, see Figure 1). The CBT app may be particularly beneficial to those patients who experience significant side effects with medication.

Strengths of this study include the concealed randomization, the successful masking of the outcome assessors, the close to 100% follow-up, the stratified randomization that allowed assessment of intervention impact in two key samples, and corroboration of the secondary outcomes including patients’ self-reports. We followed the participants for 8 weeks after the end of the randomized comparison: the participants in the active intervention arm maintained the benefits, and the participants in the control arm who had access to Kokoro-app improved. Finally, the uptake of the CBT sessions via smartphone was satisfactory, with close to 90% of the participants finishing at least six of eight program sessions and actively engaging in homework tasks.

This study is not without limitations. First, it is possible that participants in the control condition, who were not allowed access to CBT sessions at the beginning but only after the waiting period, may have suffered “disappointment effect” through the intervention period. We tried to mitigate this limitation that may be common to many waiting list-controlled trials by not making our control condition a simple waiting list control in which the participants are not allowed to change their treatment [54], but an active medication change, which is one of the recommended treatment options for antidepressant-refractory patients. Nonetheless, the possibility of some contribution of disappointment effect among the control group cannot be negated. Secondly, it was impossible to mask the participants and the clinicians administering drug treatment to the treatment allocation. However, we employed the remote telephone assessors to conduct assessments of the primary outcome, which resulted in successful masking. Lastly, it must be pointed out that in this refractory population, although the smartphone CBT in conjunction with medication switch substantively decreased depression severity, approximately 70% to 80% were still not remitted after 2 months of the combined treatment; additional face-to-face standard CBT may be helpful for these remaining patients.

These findings have demonstrated the effectiveness of smartphone CBT as an adjunctive intervention for antidepressant-resistant major depression. Given the merits of the mobile mental health intervention, including accessibility, affordability, quality control, and effectiveness, patients and clinicians may wish to use smartphone CBT as an adjunctive intervention when their depression does not respond adequately to antidepressant treatment alone. Further research of its effectiveness in wider clinical contexts, including its use as a stand-alone treatment in major depression and its role in relapse prevention, and in public health contexts such as its use for subthreshold depressive states or its utility in low- and middle-income countries is warranted. Elucidation of the effective components of the smartphone CBT package and their appropriate dosages and their integration within the existing health care systems also constitute areas needing further research.

## Appendix

Multimedia Appendix 1 Original study protocol (August 2014) and statistical analysis plan (July 2016).

Multimedia Appendix 2 FLATT investigators and committee members.

Multimedia Appendix 3 Contents of Kokoro-app.

Multimedia Appendix 4 Blinded data analyses statement of interpretation.

Multimedia Appendix 5 CONSORT‐EHEALTH checklist (V.1.6.1).

## Abbreviations

BDI-II: Beck Depression Inventory-II
CBT: cognitive behavioral therapy
FIBSER: Frequency, Intensity, and Burden of Side Effects Ratings
PHQ-9: Patient Health Questionnaire 9
RCT: randomized controlled trial
