# Correction: Smartphone Cognitive Behavioral Therapy as an Adjunct to Pharmacotherapy for Refractory Depression: Randomized Controlled Trial

**DOI:** 10.2196/11702

**Published:** 2018-08-31

**Authors:** Akio Mantani, Tadashi Kato, Toshi A Furukawa, Masaru Horikoshi, Hissei Imai, Takahiro Hiroe, Bun Chino, Tadashi Funayama, Naohiro Yonemoto, Qi Zhou, Nao Kawanishi

**Affiliations:** ^1^ Mantani Mental Clinic Hiroshima Japan; ^2^ Aratama Kokorono Clinic Nagoya Japan; ^3^ Department of Health Promotion and Human Behavior, School of Public Health, Graduate School of Medicine, Kyoto University Kyoto Japan; ^4^ National Center of Neurology and Psychiatry Kodaira Japan; ^5^ Waseda Clinic Kani Japan; ^6^ Ginza Taimei Clinic Tokyo Japan; ^7^ Funayama Mental Clinic Nagoya Japan; ^8^ Department of Biostatistics, School of Public Health, Graduate School of Medicine, Kyoto University Kyoto Japan; ^9^ Department of Health Research Methods, Evidence, and Impact, McMaster University Hamilton, ON Canada; ^10^ Advanced Telecommunications Research Institute International Kyoto Japan

The authors of “Smartphone Cognitive Behavioral Therapy as an Adjunct to Pharmacotherapy for Refractory Depression: Randomized Controlled Trial” (J Med Internet Res 2017;19(11):e373) incorrectly listed “September 2, 2015” as the starting date of patient eligibility assessment. However, “September 2, 2014” is the correct date.

The correction will appear in the online version of the paper on the JMIR website on August 31, 2018, together with the publication of this correction notice. Because this was made after submission to PubMed, Pubmed Central, and other full-text repositories, the corrected article also has been re-submitted to those repositories.

