# Supplementary material for: Smartphone Cognitive Behavioral Therapy as an Adjunct to Pharmacotherapy for Refractory Depression: Randomized Controlled Trial
Source: J Med Internet Res. 2017 Nov 3;19(11):e373. doi: 10.2196/jmir.8602 (PMC5695656; doi:10.2196/jmir.8602)
Supplement: Multimedia Appendix 2 [file jmir_v19i11e373_app2.pdf]

Multimedia Appendix 2

FLATT investigators and committee members

Steering Committee: Toshi A. Furukawa (Chair and Principal Investigator, Kyoto University), Masaru Horikoshi (National Center of Neurology and Psychiatry), Tatsuo Akechi (Nagoya City University), Shinji Shimodera (Kochi University), Mitsuhiko Yamada (National Center of Neurology and Psychiatry), Masatoshi Inagaki (Okayama University), Norio Watanabe (Kyoto University), Naohiro Yonemoto (Kyoto University), Imai Hissei (Kyoto University), Aran Tajika (Kyoto University), Yusuke Ogawa (Kyoto University), Nozomi Takeshima (Kyoto University), Yu Hayasaka (Kyoto University)

Study Statisticians: Naohiro Yonemoto (Kyoto University), Qi Zhou (McMaster University)

Data and Safety Monitoring Board: Teruhiko Higuchi (National Center of Neurology and Psychiatry), Yoshio Hirayasu (Yokohama City University), Akiko Kada (Nagoya Medical Center)

Site Principal and Co-principal Investigators: **Hokkaido University**: Ichiro Kusumi, Yuki Kako, **Toho University**: Masafumi Mizuno, Naohisa Tsujino, **Nagoya City University**: Tatsuo Akechi, Sei Ogawa, **Hiroshima University**: Shigeto Yamawaki, Yasumasa Okamoto, Ran Jinnin, **Kochi University**: Shigeru Morinobu, Shinji Shimodera, Hirokazu Fujita

Participating Clinical Sites (number of participants recruited):
Hokkaido University (12): Nobuki Kitagawa (Hokudaidori Kokorono Clinic), Yuki Kako (Hokkaido University), Hisashi Narita (Hokkaido University), Yoshihito Takahashi (Souen Mental Clinic)
Toho University (22): Bun Chino (Ginza Taimei Clinic), Naohisa Tsujino (Toho University), Hiroko Hasuya (Toho University)
Nagoya City University (75): Tadashi Kato (Aratama Kokorono Clinic), Takahiro Hiroe (Waseda Clinic), Tadashi Funayama (Funayama Mental Clinic), Yoshio Ikeda (Narumi Himawari Clinic), Yoshihiro Shinagawa (Shiki Clinic), Masaki Kondo (Aratama Kokorono Clinici/Nagoya City University), Taku Sugiura (Aratama Kokorono Clinic/Inuyama Hospital), Toshitaka Ii (Nagoya City University), Nao Shiraishi (Nagoya City University)
Hiroshima University (39): Akio Mantani (Mantani Mental Clinic), Yasutaka Fujita (Kusatsu Hospital), Keigo Nakatsu (Kusatsu Hospital), Takahiro Miyazaki (Kusatsu Hospital), Ran Jinnin (Hiroshima University), Atsuo Yoshino (Hiroshima University)
Kochi University (17): Shinji Shimodera (Kochi University), Hirotoshi Sato (Harimayabashi Clinic), Hirokazu Fujita (Kochi University)
