# Supplementary material for: Smartphone Cognitive Behavioral Therapy as an Adjunct to Pharmacotherapy for Refractory Depression: Randomized Controlled Trial
Source: J Med Internet Res. 2017 Nov 3;19(11):e373. doi: 10.2196/jmir.8602 (PMC5695656; doi:10.2196/jmir.8602)
Supplement: Multimedia Appendix 3 [file jmir_v19i11e373_app3.pdf]

Multimedia Appendix 3

Contents of Kokoro-app

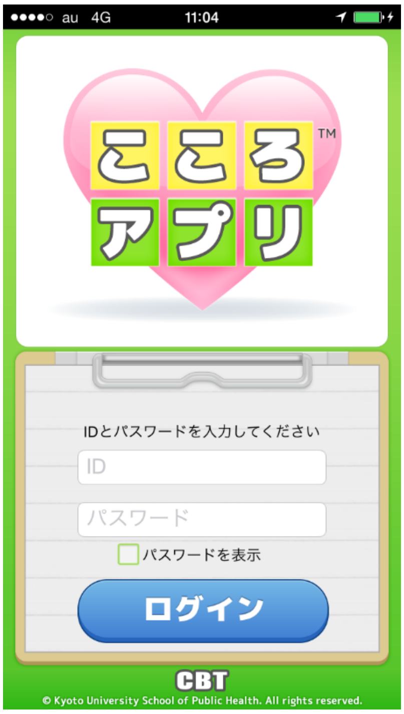

This is the log-in page.
The patient enters his/her ID and password, and provides some preliminary information such as his/her alias figure and his/her alias name.

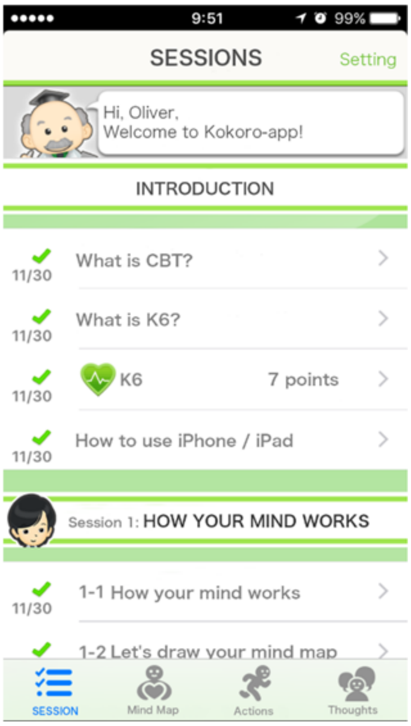

This is the main page, showing session names and their subsections.

The buttons at the bottom will lead the patient to the main page [SESSIONS], a page for self-monitoring by filling in a mind map [Mind Map], a page for planning and executing a behavioural activation "personal experiment" [Actions], or a page for devising alternative thoughts [Thoughts].

At the beginning of each session, a window for K6 pops up for the patient to self-monitor his/her current distress level.

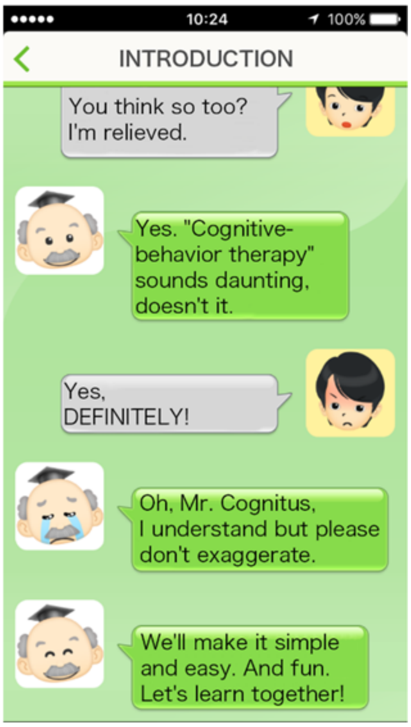

Each session contains dialogues between characters (Professor Kokoro, Mr Cognitus, Ms Actie, Ms Assertia, CBT Robot, Junk Robot) who explain the principles and skills of CBT.
Altogether there are 8 sessions: one introduction, two on self-monitoring, two on behavioral activation, two on cognitive restructuring, and one epilogue.

#	Title	Contents
Introduction		Intro to CBT<br>Training in iPhone
Session 1	Mind map (1)	Cognitive-behavioral model
Session 2	Mind map (2)	Tips: Emotion as alarm, Slice of time, Habituation
Session 3	Behavioral activation (1)	"When the body movers, so does the mind"
Session 4	Behavioral activation (2)	Tips: Start small and near
Session 5	Cognitive restructuring (1)	(1) fact glasses<br>(2) % calculator
Session 6	Cognitive restructuring (2)	(3) friend's call<br>(4) what now microphone
Epilogue		Summary<br>Relapse prevention<br>Trouble shooting for the future

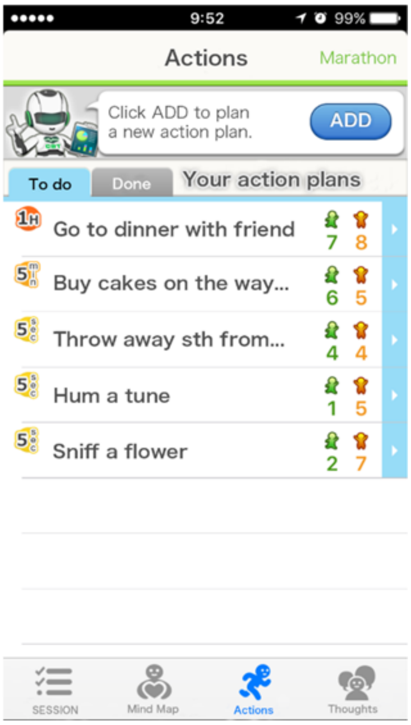

This is a page for planning a behavioral activation "personal experiment." They are called and explained in the Sessions as "personal experiment" to emphasize two aspects. First, each behavior may or may not be fun for each person. The patient therefore has to find it out for himself personally. Second, the patient will have to observe what happens to him as he performs, or tries to perform, the action, as if it were a true experiment.

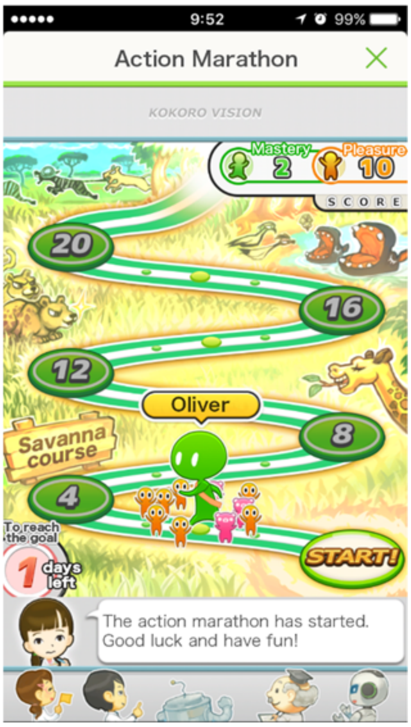

When the patient completes his/her behavioral experiment, the app opens up a page for “Action Marathon,” where the patient’s alias can advance the stages depending on how many points of pleasure and mastery he/she has found.

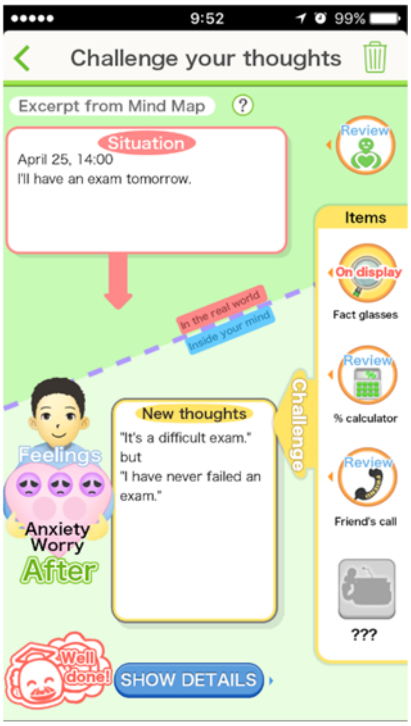

This is a page for cognitive restructuring. Each of the four items to the right of the display opens up a dialogue with the “item” which will lead the patient through Socratic questions to alternative thoughts.
