# Supplementary material for: Smartphone Cognitive Behavioral Therapy as an Adjunct to Pharmacotherapy for Refractory Depression: Randomized Controlled Trial
Source: J Med Internet Res. 2017 Nov 3;19(11):e373. doi: 10.2196/jmir.8602 (PMC5695656; doi:10.2196/jmir.8602)
Supplement: Multimedia Appendix 4 [file jmir_v19i11e373_app4.pdf]

Multimedia Appendix 4

Blinded data analyses statement of interpretation

FLATT trial: Blinded Data Analyses Statement of Interpretation

February 6, 2017

The members of the writing committee of the FLATT trial (undersigned below) developed and recorded two interpretations of the results on the basis of a blinded review of the statistical analyses (efficacy comparison comparing treatment A compared with Treatment B, and effectiveness comparison comparing treatment AA compared with Treatment BB), with one assuming that A was the smartphone CBT + antidepressant switch arm and B was the antidepressant switch alone arm, and another assuming vice versa.

A = smartphone CBT + antidepressant switch arm and B = antidepressant switch alone arm

1 In the efficacy comparison A vs B,
   1.1 There is no important baseline imbalance.
   1.2 In terms of the primary outcome, the adjusted analysis shows that A is superior to B by 1.72 points (95%CI: -3.18 to -0.25, p=0.02) on mean PHQ-9 at week 9.
   1.3 In terms of the secondary outcomes of efficacy, A was barely superior to B in terms of mean BDI-II (difference=-3.17, (-6.34, 0.01), 0.05), or not superior to B in remission rates (OR=1.92 (0.72 to 5.14), p=0.19) and in mean treatment satisfaction (difference=9.4 (-3.2, 22.1), 0.14) at week 9.
   1.4 Patients in treatment A reported significantly less mean burden of side effects (FIBSER difference -0.75 (-1.47 to -0.03), p=0.04) than those in treatment B. Two persons each in the two groups discontinued antidepressant treatment by week 9. There was one report of suicidality (self-injurious behavior without suicidal intent according to C-CASA) in A and one report of a serious adverse event in B (brief hospital admission for examination of pre-existing spinal canal stenosis).
2 In the effectiveness comparison AA vs BB,
   2.1 There is no important baseline imbalance.
   2.2 In terms of the primary outcome, the adjusted analysis shows that AA is superior to BB by 2.48 points (95%CI: -3.72, -1.23 p=0.0001) on mean PHQ-9 at week 9.
   2.3 In terms of the secondary outcomes of efficacy, AA is superior to BB in terms of mean BDI-II (difference=-4.05, (-6.63, -1.48), 0.002), or mean treatment satisfaction (difference=16.3 (5.7, 26.8), 0.003). The remission rates were not significantly different (difference=14.1% (-2.3, 30.4), 0.06).
   2.4 With regard to harm outcomes, there was no significant differences between AA and BB in terms of mean FIBSER (-0.76, (-1.58, 0.05), 0.07). Five in AA and two in BB discontinued antidepressant treatment by week 9. There was one report of suicidality (self-injurious behavior without suicidal intent according to C-CASA) in A and one report of a serious adverse event in B (brief hospital admission for examination of pre-existing spinal canal stenosis).
3 To summarize,
   3.1 The addition of smartphone CBT to antidepressant switch among patients with refractory depression led to an effect size of about 0.3-0.4 in both the efficacy and effectiveness comparisons, corresponding with an NNT of about 7-11.
   3.2 The above efficacy and effectiveness findings of the primary outcome were largely corroborated by various secondary outcomes of efficacy.
   3.3 Moreover, there was indication that CBT reduced the global burden of side effects, although the amount of administered drugs were similar between the two groups (e.g. 9.7 mg/day vs 10.1 mg/day for escitalopram (p=0.56) and 81.6 mg/day vs 83.0 mg/day for sertraline (p=0.86) in the efficacy comparison). And there were no important differences in any other harm outcomes, including suicidality or serious adverse events.
   3.4 After week 9 when the randomized comparison ended, the treatment effect was maintained for those who initially received smartphone CBT after 9 weeks up to 17 weeks, while the addition of smartphone CBT after 9 weeks of medication switch resulted in symptom

reduction less pronounced but similar to that seen in the arm with immediate smartphone CBT use up to week 9.

3.5 Bang Index indicated that either "blinded rating" or "wishful rating" were dominant across two arms (the raters guessed the treatment assignment haphazardly or guessed it to be smartphone CBT arm more often regardless of the actual assignment), resulting in ideally unbiased assessment of outcomes in the trial.

4 To conclude,

4.1 Given the accessibility and affordability of smartphone CBT, it is clinically worthwhile to consider adding smartphone CBT to antidepressant therapy in patients with major depression, especially when they do not respond to antidepressant therapy alone.

A = antidepressant switch alone arm and B= smartphone CBT + antidepressant switch arm

1 Same as above

2 Same as above

3 To summarize,

3.1 The addition of smartphone CBT to antidepressant switch among patients with refractory depression hampered the recovery process that would have happened with medication switch alone, both in the efficacy or effectiveness samples.

3.2 By using smartphone CBT, patients felt greater burden of side effects. Serious adverse events, however, did not differ between the two groups.

3.3 When the patients stopped using the smartphone CBT, the patients started to ameliorate, although not as completely as they would have, had they not been asked to use smartphone app from the beginning.

3.4 The assessment was ideally blinded, with little indication of bias due to unblinding of the assessors.

4 To conclude,

4.1 Smartphone CBT appears to hamper recovery and increase side effect burdens among patients with major depression treated with antidepressant therapy. Smartphone CBT is not recommended for this population.

Tatsuo Akechi   Mitsuhiko Yamada   Shinji Shimodera

Masaru Horikoshi   Hissei Imai   Masatoshi Inagaki

Norio Watanabe   Yusuke Ogawa   Aran Tajika
